# Risk and determinants of mortality associated with invasive group A streptococcus (iGAS) disease in Scotland: A national surveillance study

**DOI:** 10.1016/j.ijid.2025.108090

**Published:** 2025-12

**Authors:** Ebrahim Ghaderi, Eisin McDonald, Hazel Henderson, Melissa Llano, Diane Lindsay, Morris C. Muzyamba, Navneet Rai, Peter MacPherson

**Affiliations:** 1 Respiratory Bacterial Pathogens Team, Public Health Scotland, Glasgow, Scotland; 2 Scottish Microbiology Reference Laboratory, Glasgow Royal Infirmary, Glasgow, Scotland; 3 School of Health and Wellbeing, University of Glasgow, Glasgow, United Kingdom; 4 Faculty of Infectious and Tropical Disease, London School of Hygiene and Tropical Medicine, London, United Kingdom

**Keywords:** Group A Streptococcus, Epidemiology, Surveillance, Mortality, Risk factors

## Abstract

**Objectives:**

Understanding determinants of mortality in invasive Group A Streptococcal (iGAS) infections is essential for developing targeted public health measures and improving clinical management. Using a comprehensive prospectively-collected national surveillance dataset, we set out to investigate factors associated with case fatality in Scotland.

**Methods:**

We included all episodes of iGAS notified in Scotland surveillance data from January 2017 to December 2024. Case fatality was ascertained by 7-day follow-up. Regression models incorporating key clinical, microbiological, and demographic predictors were constructed to investigate factors associated with higher case fatality.

**Results:**

There were 2209 iGAS notifications, of whom 191 died (8.6%). In multivariate analysis, increasing age (OR for 45-64 years: 2.36 [95% CI:1.3-4.48]; OR for 65-74 years: 4.54 [2.41-8.89]; and OR for 75 years and over: 7.77 [3.4-14.8]; emm type 3.93 (OR: 3.13 [1.43-6.76]) and emm type 1 (3.44 [2-6.15]); alcohol misuse (2.56 [1.36-4.63]); respiratory tract infection (2.07 [1.27-3.32]); and intensive care admission (2.5 [1.7-3.66]) were all significantly associated with mortality.

**Conclusion:**

Case fatality from iGAS was high, particularly for vulnerable populations, and for those with emm type 3.93 and emm type 1 infections. Novel clinical, therapeutic, and public health interventions to improve clinical outcomes are required.

## Highlights

• Increasing age is a major risk factor for mortality in iGAS.
• Certain emm types, particularly emm type 1 and emm type 3.93, are significantly associated with higher CFR.
• Alcohol misuse and respiratory infections were identified as significant factors to iGAS mortality.

## Introduction

Invasive Group A Streptococcal (iGAS) infections caused by the bacterium *Streptococcus pyogenes* can infect sterile sites within the body [1], causing significant clinical and public health concern due to disease severity and high mortality. Severe clinical manifestations include necrotizing fasciitis, streptococcal toxic shock syndrome, and bacteraemia, often requiring prolonged, intensive care and rehabilitation [2,3]. The incidence of iGAS follows a seasonal pattern, particularly peaking in spring, and often affects vulnerable populations, including those with underlying health conditions [2,4].

Several demographic and clinical factors have been identified as important contributors to increased risk of death. Age is a critical factor, with both the very young and the elderly being at increased risk, and underlying health conditions such as diabetes, heart disease, and malignancies have been associated with high mortality [5]. Socioeconomic factors also play a role, with individuals from lower socioeconomic backgrounds – who may experience barriers to diagnosis and timely medical care – having worse outcomes [6]. Identifying risk factors for death in iGAS is useful to both clinicians and public health policymakers, as there are limited current effective prevention approaches [7].

Recent studies have indicated that the emergence of specific strains of *Strep. pyogenes* has been linked to increased virulence and higher rates of severe disease [8]. iGAS has several virulence factors, including the M protein, which consists of numerous *emm* types that vary in distribution by location and through time [9]. The relationship between *emm* type and mortality in iGAS is not clearly understood but certain *emm* types are often more associated with invasive disease, for example, *emm* 1 [10]*.*

Understanding the multifaceted factors contributing to mortality from iGAS is essential for developing targeted public health interventions and improving clinical management strategies. Using comprehensive national surveillance data and linked mortality records, we therefore set out to investigate the determinants of case fatality among people notified with iGAS in Scotland.

## Material and methods

### iGAS surveillance in Scotland

In Scotland, iGAS infections are defined as isolation of Group A Streptococcus (GAS) from a normally sterile site by culture or polymerase chain reaction (PCR). iGAS samples are forwarded to the Scottish Microbiology Reference Laboratory (SMiRL) for further analysis and typing. We included all notifications of iGAS in Scotland surveillance data from 1st January 2017 to 31st December 2024. Surveillance of iGAS infections relies on routine laboratory data, and these laboratory isolates are subject to notification requirements under the Public Health, etc. (Scotland) Bill 2008 [11]. An iGAS confirmed case is defined as a case with a severe clinical presentation consistent with iGAS or severe GAS infection, plus laboratory criteria: isolation of group A streptococcus by culture or molecular methods from a normally sterile body site. Records of all culture and PCR-positive iGAS samples from laboratories across Scotland are submitted to Public Health Scotland (PHS) through the Electronic Communication of Surveillance in Scotland (ECOSS) system. Additionally, an enhanced surveillance questionnaire is completed by local public health teams to gather detailed information about clinical presentations, risk factors and patient outcomes through a reviewing of medical files and interviews with the patients.

#### Ascertainment of patient outcomes

A death related to iGAS was defined as a death from any cause occurring within seven days of iGAS confirmation. PHS conducts active follow-ups on the outcomes of all reported cases 7 days to ensure comprehensive data capture. This was accomplished through a process of active follow-up by consultation with health protection teams and checking medical registers for outcomes, which were then recorded in the surveillance data.

### Ethics statement

In accordance with the Public Health etc. (Scotland) Act 2008, SMiRL and PHS are required to process data related to notifiable diseases and organisms, as well as conduct public health investigations, meaning that individual patient consent was not necessary. We have not included any identifiable patient data in this paper.

### Statistical analysis

R version 4.1.2 was used for the analysis. Descriptive statistics, including frequency and percentages, were used to summarise participant characteristics. In univariate analysis, associations between death at seven days and clinical, microbiological, and demographic risk factors were examined using the Chi-square and Fisher’s exact tests. In multivariable modelling, our primary intention was to investigate the magnitude of association between strain type and case fatality, after adjusting for clinical and demographic confounders. Then in secondary analysis, we additionally investigated the adjusted effects of covariates on case fatality. Two logistic regression models were developed to investigate the relationship between death and other factors: one model included intensive treatment unit (ITU) admission as a covariate and the other did not (to remove the interactions between ITU admission and other variables, including comorbidities, and check the similarity of the results). A *P*-value of less than 0.05 was considered significant.

## Results

**Figure 1:**
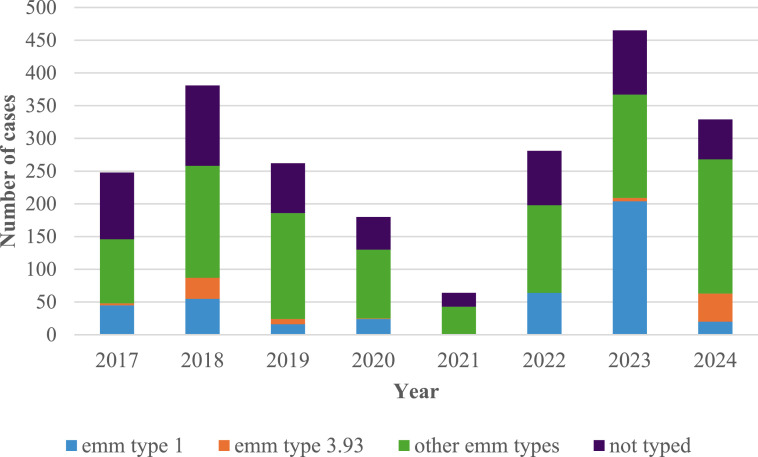
Annual numbers of iGAS notifications by *emm* type from 2017 to 2024 in Scotland.

During the eight-year study period, 2209 iGAS case had been notified. The clinical samples were categorized as follows: blood samples (1622; 73.4%), other normally sterile sites (441; 20%), respiratory samples (77; 3.5%), joint aspiration (61; 2.8%), and cerebrospinal fluid (CSF) (8; 0.3%). In total, 1,522 cases (68.4%) had bacteraemia. The majority of iGAS cases (2,163; 97.9%) were evaluated using culture. The highest number of cases was 465 in 2023, and the prevalence of various *emm* types isolated varied across the years (Figure 1).

There were 1192 males (54%) and 1017 females (46%) with no significant difference in the distribution of the various *emm* types between the sexes (*P* = 0.989). The most common age group was 15-44 years (604 cases; 27.3%), followed by 45-64 years (590 cases; 26.7%). The distribution of various *emm* types among age groups was significantly different (*p*-value <0.001, Table 1).

**Table 1:** Distribution of *emm* types by age group and sex.

Variable	*emm* type 1 Frequency (%)	*emm* type 3.93 Frequency (%)	Other *emm* types Frequency (%)	Not typed Frequency (%)	Total Frequency (%)	*P*-value
**Age group**						
0-14	92 (29.9%)	17 (5.5%)	120 (39%)	79 (25.6%)	308 (100%)	<0.001*
15-44	65 (10.8%)	17 (2.8%)	309 (51.2%)	213 (35.3%)	604 (100%)	
45-64	118 (20%)	21 (3.6%)	285 (48.3%)	166 (28.1%)	590 (100%)	
65-74	71 (21.8%)	20 (6.2%)	153 (47.1%)	81(24.9%)	325 (100%)	
75 and more	79 (20.7%)	15 (3.9%)	220 (57.6%)	68 (17.8%)	382 (100%)	
**Sex**						
Female	196 (19.3%)	42 (4.1%)	503 (49.5%)	276 (27.1%)	1017 (100%)	0.989
Male	229 (19.2%)	48 (4%)	584 (49%)	331 (27.8%)	1192 (100%)	

Chi-square test was applied for analysis.

⁎ Statistically significant differences.

**Figure 2:**
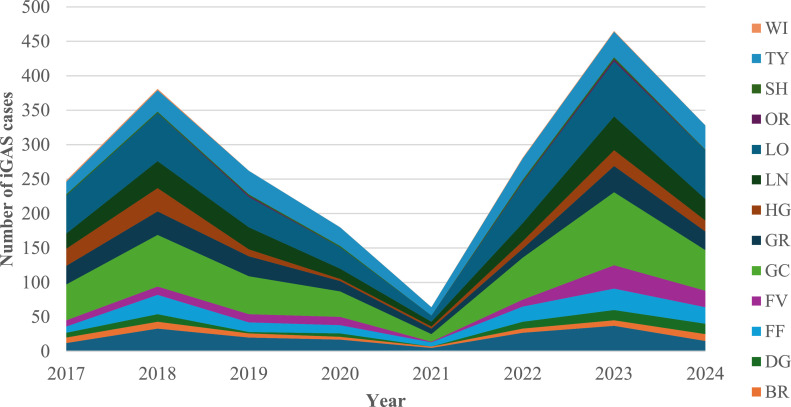
Distribution of iGAS notifications by NHS boards from 2017 to 2024. NHS boards; AA: Ayrshire & Arran, BR: Borders, DG: Dumfries & Galloway, FF: Fife, FV: Forth Valley, GGC: Greater Glasgow & Clyde, GR: Grampian, HG: Highland, LN: Lanarkshire, LO: Lothian, OR: Orkney, SH: Shetland, TY: Tayside

The highest number of iGAS cases was reported from NHS Greater Glasgow and Clyde (20.6%, 456/2209), followed by NHS Lothian (19%, 419/2209) (Figure 2).

**Figure 3:**
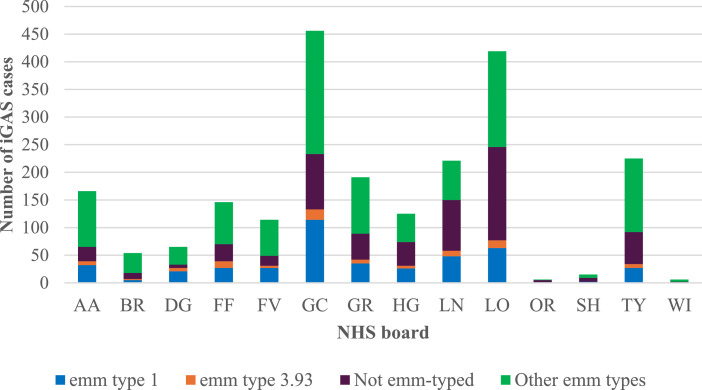
Distribution of *emm* types by NHS Scotland health boards from 2017 to 2024. NHS boards; AA: Ayrshire & Arran, BR: Borders, DG: Dumfries & Galloway, FF: Fife, FV: Forth Valley, GGC: Greater Glasgow & Clyde, GR: Grampian, HG: Highland, LN: Lanarkshire, LO: Lothian, OR: Orkney, SH: Shetland, TY: Tayside.

In terms of geographical *emm* type distribution the highest proportion of *emm* type 1 was found in NHS Dumfries & Galloway (32.3%, 21/65), followed by NHS Greater Glasgow and Clyde (25%, 114/456) and Forth Valley (23.7%, 27/114). The highest proportion of *emm* type 3.93 was also in NHS Dumfries & Galloway (9.2%, 6/65), followed by NHS Fife (8.2%, 12/146) and Lanarkshire (4.5%, 10/221) (Figure 3).

### Univariate analysis

The overall case fatality rate (CFR) was 8.6% (191/2209). In this study, there was no significant difference in CFR between males (92/1192, 7.7%) and females (99/1017, 9.7%; *p* = 0.11). There was a significant relationship between death and age group (*p* < 0.001). Those aged 75 years and older had the highest CFR (68/383; 17.7%), while those aged 15-44 years had the lowest CFR (18/604; 3%). *emm* type 3.93 had a higher CFR (19/93; 20.4%) than *emm* type 1 (77/428; 18%) and other *emm* types (75/1075; 7%; *p* < 0.001).

Other significant factors related to increased mortality risk included bacteraemia (p<0.001), chronic cardiac disease (*p* = 0.001), diabetes (*p* = 0.022), malignancy (*p* = 0.046), respiratory tract infection (*p* = 0.005), and admission to ITU (*p* < 0.001). Conversely, surgical wounds (*p* = 0.003), skin lesion wound (*p* = 0.01), persons who inject drugs (PWID) (*p* < 0.001), septic arthritis (*p* < 0.001), sore throats (*p* = 0.002) and antibiotic resistance (*p* = 0.005) were negatively related to death (Table 2).

**Table 2:** Frequency and percentage of patient characteristics and univariate analysis.

Variable	Did not die Frequency (%)	Died Frequency (%)	Total Frequency (%)	*P*-value
**Age group** (n = 2209)				
0-14	290 (94.2%)	18 (5.8%)	308 (13.9)	<0.001*
15-44	586 (97)	18 (3%)	604 (27.3)	
45-64	543 (92%)	47 (8%)	590 (26.7)	
65-74	286 (88%)	39 (12%)	325 (14.7)	
75 and more	314 (82%)	69 (18%)	382 (17.3)	
**Sex** (n = 2209)				
Male	1100 (92.3%)	92 (7.7%)	1192 (54)	0.11
Female	919 (90.3%)	99 (9.7%)	1017 (46)	
**emm type** (n = 2209)				
Not typed	594 (96.7%)	20 (3.3%)	608 (27.5)	<0.001*
Other type	1000 (93%)	75 (7%)	1086 (49.2)	
1	351 (82%)	77 (18%)	425 (19.2)	
3.93	74 (79.6%)	19 (20.4%)	90 (4.1)	
**Bacteraemia** (positive blood sample) (n = 2209)				
No	679 (97.4%)	18 (2.6%)	697 (31.6)	<0.001*
Yes	1339 (88.6%)	173 (11.4%)	1512 (68.4)	
**Alcohol misuse** (n = 1662)				
No	1383 (89.1%)	170 (10.9%)	1553 (93.4)	0.062
Yes	91 (82.7%)	19 (17.3%)	110 (6.6)	
**Surgical wound** (n = 1660)				
No	1370 (88%)	187 (12%)	1557 (93.7)	0.003*
Yes	102 (98.1%)	2 (1.9%)	104 (6.3)	
**Skin lesion wound** (n = 1668)				
No	972 (87.2%)	143 (12.8%)	115 (66.8)	0.01*
Yes	507 (91.5%)	47 (8.5%)	554 (33.2)	
**Chronic cardiac disease** (n = 1660)				
No	1333 (89.5%)	156 (10.5%)	1489 (89.6)	0.001*
Yes	139 (80.8%)	33 (19.2%)	172 (10.4)	
**PWID** (n = 1668)				
No	1274 (87.4%)	183 (12.6%)	1457 (87.3)	<0.001*
Yes	205 (96.7%)	7 (3.3%)	212 (12.7)	
**Septic arthritis** (n = 1660)				
No	1359 (87.8%)	188 (12.2%)	1547 (93.1)	<0.001*
Yes	113 (99.1%)	1 (0.9%)	114 (6.9)	
**Pneumonia** (n = 1663)				
No	1297 (89.1%)	159 (10.9%)	1456 (87.5)	0.169
Yes	178 (85.6%)	30 (14.4%)	208 (12.5)	
**Chronic pulmonary disease** (n = 1662)				
No	1387 (88.9%)	174 (11.1%)	1561 (93.9)	0.349
Yes	87 (85.3%)	15 (14.7%)	102 (6.1)	
**Diabetes** (n = 1661)				
No	1292 (89.3%)	154 (10.7%)	1446 (87)	0.022*
Yes	181 (83.8%)	35 (16.2%)	216 (13)	
**Immunosuppression** (n = 1660)				
No	1372 (88.7%)	174 (11.3%)	1546 (93.1)	0.476
Yes	99 (86.1%)	16 (13.9%)	115 (6.9)	
**Malignancy** (n = 1660)				
No	1384 (89.1%)	170 (10.9%)	1554 (93.6)	0.046*
Yes	88 (82.2%)	19 (17.8%)	107 (6.4)	
**Sore throat** (n = 1660)				
No	1253 (87.6%)	177 (12.4%)	1430 (86.1)	0.002*
Yes	219 (94.8%)	12 (5.2%)	231 (13.9)	
**Respiratory tract infection** (n = 1663)				
No	1328 (89.4%)	157 (10.6%)	1485 (89.2)	0.005*
Yes	147 (82.1%)	32 (17.9%)	179 (10.8)	
**Steroid use** (n = 1660)				
No	1436 (88.8%)	182 (11.2%)	1618 (97.4)	0.434
Yes	36 (83.7%)	7 (16.3%)	43 (2.6)	
**ITU admission** (n = 1679)				
No	1185 (90.7%)	121 (9.3%)	1306 (81.7)	<0.001
Yes	309 (82.6%)	65 (17.4%)	374 (22.3)	
**IVIG** (n = 1675)				
No	1424 (88.6%)	184 (11.4%)	1608 (95.9)	0.636
Yes	62 (91.2%)	6 (8.8%)	68 (4.1)	
**Antibiotic resistance** (n = 2209)				
No resistance	1567 (90.4%)	167 (9.6%)	1734 (66.7)	0.005*
≤ 2 antibiotic resistances	370 (94.6%)	21 (5.4%)	391 (15)	
≥ 3 antibiotic resistances	82 (96.5%)	3 (3.5%)	476 (18.3)	

Chi-square test was applied for analysis.

⁎ Statistically significant differences. ITU: Intercave treatment unit CI: Confidence interval PWID: Person who injects drugs IVIG: Intravenous immunoglobulin

### Multivariate analysis

In our first model (ITU included), older age (OR from 2.36-7.77), *emm* type 3.93 (OR: 3.13; CI_95%_:1.43-6.76) and *emm* type 1 (OR: 3.44; CI_95%_: 2-6.15); alcohol misuse (OR: 2.56; CI_95%_: 1.36-4.63), respiratory tract infection (OR: 2.07; CI_95%_: 1.27-3.32), and intensive care admission (OR: 2.5; CI_95%_: 1.7-3.66) were all significantly associated with increased odds of case fatality. Age 0-14 years, diabetes, and malignancy were not significantly associated with death. Surgical wound (OR: 0.15; CI_95%_: 0.02-0.51), skin lesion wound (OR: 0.62; CI_95%_: 0.42-0.92), septic arthritis (OR: 0.1; CI_95%_: 0-0.46), and sore throat (OR: 0.26; CI_95%_: 0.13-0.5) were remained inversely associated with case fatality (Table 2). The results from the second multivariate model (ITU excluded) were very similar to those of the first model; however, the variable for sex became non-significant (see Table 3).

**Table 3:** Multivariate analysis of risk factors for death in iGAS cases.

Variables (baseline)	Multivariate analysis		Multivariate analysis (ITU admission excluded)		Multivariate analysis (Bacteraemia included)	
	Odds ratio (95%CI)	*P*-Value	Odds ratio (95%CI)	*P*-Value	Odds ratio (95%CI)	*P-*Value
**Sex** (male)						
Female	1.42 (1.01-1.99)	0.042*	1.36 (0.98-1.9)	0.064	1.42 (1.01-1.99)	0.042*
**Age group** (15-44 years)						
0-14	1.74 (0.81-3.7)	0.147	1.78 (0.86-3. 7)	0.117	1.74 (0.81-3.7)	0.147
45-64	2.36 (1.3-4.48)	0.006*	2.7 (1.5-5.05)	0.001*	2.36 (1.3-4.48)	0.006*
65-74	4.54 (2.41-8.89)	<0.001*	4.25 (2.3-8.2)	<0.001*	4.54 (2.41-8.89)	<0.001*
75 and older	7.77 (4.3-14. 8)	<0.001*	6.73 (3.8-12.57)	<0.001*	7.77 (4.3-14. 8)	<0.001*
***emm* type** (not typed)						
Other type	1.27 (0.75-2.26)	0.38	1.14 (0.68-2)	0.614	1.27 (0.75-2.26)	0.38
1	3.44 (2-6.15)	<0.001*	3.33 (1.96-5.87)	<0.001*	3.44 (2-6.15)	<0.001*
3.93	3.13 (1.43-6.76)	<0.001*	3.84 (1.85-8)	<0.001*	3.13 (1.43-6.76)	<0.001*
**Bacteraemia** (positive blood sample) (no)	Excluded manually		Excluded manually		Removed by model	
**Alcohol misuse**	2.56 (1.36-4.63)	0.002*	2.67 (1.45-4.74)	0.001*	2.56 (1.36-4.63)	0.002*
**Surgical wound**	0.15 (0.02-0.51)	0.012*	0.16 (0.02-0.56)	0.015*	0.15 (0.02-0.51)	0.012*
**Skin lesion wound**	0.62 (0.42-0.92)	0.018*	0.58 (0.39-0.85)	0.006*	0.62 (0.42-0.92)	0.018*
**Chronic cardiac disease**	Removed by model		Removed by model		Removed by model	
**PWID**	Removed by model		Removed by model		Removed by model	
**Septic arthritis**	0.1 (0-0.46)	0.023*	0.08 (0-0.38)	0.014*	0.1 (0-0.46)	0.023*
**Pneumonia**	Removed by model		Removed by model		Removed by model	
**Chronic pulmonary disease**	Removed by model		Removed by model		Removed by model	
**Diabetes**	1.53 (0.96-2.4)	0.006*	1.39 (0.88-2.15)	0.148	1.53 (0.96-2.4)	0.006*
**Immunosuppression**	Removed by model		Removed by model			
**Malignancy**	1.7 (0.94-2.97)	0.07	Removed by model		1.7 (0.94-2.97)	0.07
**Sore throat**	0.26 (0.13-0.5)	<0.001*	0.29 (0.14-0.54)	<0.001*	0.26 (0.13-0.5)	<0.001*
**Respiratory tract infection**	2.07 (1.27-3.32)	0.003*	1.98 (1.22-3.16)	0.005*	2.07 (1.27-3.32)	0.003*
**Steroid use**	Removed by model		Removed by model		Removed	
**ITU admission**	2.5 (1.7-3.66)	<0.001*	Excluded manually	-	2.5 (1.7-3.66)	<0.001*
**IVIG**	Removed by model		Removed by model		Removed by model	
**Antibiotic resistance**	Removed by model		Removed by model		Removed by model	

ITU: Intercave treatment unit

CI: Confidence interval

PWID: Person who injects drugs

IVIG: Intravenous immunoglobulin

⁎ Statistically significant differences.

## Discussion

The main findings from this national surveillance study conducted over 8 years in Scotland was that case fatality was persistently high (8.6% overall), and that vulnerable populations with iGAS had substantially worse outcomes. Moreover, isolation of iGAS *emm* (particularly 1 and 3.93) were strongly associated with mortality in adjusted analysis, indicating type-specific pathogenic processes that might be amenable to novel therapeutics or targeted vaccination approaches.

The relationship between iGAS *emm* type and mortality has been controversial worldwide due to various confounders and challenges within the iGAS surveillance systems. In this study, we used high-quality data from a comprehensive national surveillance system over an 8-year period and with a large sample size, facilitating better understanding of the relationship between *emm* types in iGAS and mortality, leading to more robust conclusions. In our study, the overall CFR was 8.6%. We identified a significant statistical relationship between age, *emm* types 1 and 3.93, alcohol misuse, respiratory tract infections, and admission to the ITU to mortality in the multivariate analysis. The CFR increased with age: it was 3% for individuals aged 15 to 45 years, 5.8% for those younger than 15 years, and rose to 18% for individuals aged 75 years and older. Therefore, it is essential to consider elderly close contacts in early detection activities or updating the high-risk group definition in iGAS guideline (it is ≥75 years now) to initiate antibiotics for prophylaxis, along with implementing other care measures such as infection control processes in healthcare settings to prevent mortality.

CFRs reported in other studies range from 4% to 32%, depending on the study period, study settings, age distribution, prevalence of underlying comorbidities, and available care resources. While the CFR generally tends to increase with age, the age group under 15 years is an exception, showing a higher CFR compared to middle-aged individuals [4,[12], [13], [14], [15], [16]]. The CFR in males was higher than females in Alberta (not statistically significant), and the highest CFR was seen in age ≥60 years [14]; however, in our study, females had a higher CFR than males, but it was statistically borderline significant. Additionally, the CFR appears to be higher among residents of care homes due to underlying diseases and advanced age [4,12,13]. Our study demonstrated a gradual increase in the risk of death with advancing age. The OR for age and death were 3.7 for individuals aged 50-64 years and 4.6 for those over 65 years, according to O’Loughlin et al [4]. In the study by Nelson et al., the OR was 5.4 for individuals over 75 years and 3.34 for those aged 65-74 years.^13^ This suggests that increasing age is a significant risk factor for death in iGAS, therefore elderly people should be prioritized in close contact investigation and prophylaxis.

Our study demonstrated that *emm* types are associated with increased mortality after adjusting for other factors. Specifically, *emm* type 1 had an OR of 3.44, while *emm* type 3.93 had an OR of 3.13 compared to not typed, which served as the reference category. This is an important result that requires further investigation using molecular methods to identify the basic pathophysiology. A small number of previous studies have found that *emm* types 1, 3, and 12 were associated with higher CFRs [4,12,13,16]. The OR was higher for *emm* type 3 (OR: 2.2) and *emm* type 1 (OR: 1.9) compared to other types, according to O’Loughlin et al.^4^ However, another study reported no relationship between mortality and *emm* type 3.93 in a 7-day active follow-up [17]. These findings suggest that greater understanding of the pathophysiological basis of increased mortality risk from specific *emm* types is required, and targeted surveillance and typing of *emm* types in clinical settings could enhance early detection and treatment strategies. Public health policies should use *emm* type data in the decision-making to identify at-risk populations and resources allocation.

We also found that systemic signs and symptoms were related to a higher CFR in iGAS disease without adjustment for confounders [14]. In our study, surgical wounds, skin lesions, septic arthritis, and sore throat were associated with a lower CFR, while respiratory tract infections were linked to a higher CFR. Rudolph et al [16] reported a higher CFR in endocarditis but a lower CFR in cellulitis. In another study, no relationship was found between age and death; however, a relation was identified between death and conditions such as bactaeremia, pneumonia, necrotizing fasciitis, and streptococcal toxic shock syndrome (STSS), as well as with *emm* types 1, 3, and 12, particularly in females with congestive heart failure [12]. Additionally, another study identified independent associations between specific signs and symptoms and death, including recent surgery, septic shock, necrotizing fasciitis, meningitis, isolated bacteraemia, and pneumonia [13], which have increased the mortality chance. This underscores the critical need for clinicians to recognize these systemic signs and symptoms early, as they may indicate a higher risk of mortality and necessitate timely intervention.

Regarding underlying conditions, there are a limited number of previous studies available for comparison. Based on our study, chronic cardiac disease, people who inject drugs (PWID), pneumonia, chronic pulmonary disease, and immunosuppression were not associated with death. In contrast, alcohol misuse, respiratory tract infections, and admission to an ITU were significantly related to mortality. Langley et al. [7] showed that while diabetes was linked to iGAS incidence, it did not correlate with death. They identified heart disease, malignancy, and alcohol misuse as factors related to mortality. Other studies found that underlying chronic illness and immunosuppression were associated with death [4,13]. In our study, several underlying conditions and diseases were associated with death only in univariate analysis, suggesting the effect of confounding factors that were removed in the multivariate analysis. This highlights that age, *emm* type, alcohol consumption, and consideration for ITU admission are strongly related to mortality in iGAS and should be considered in both disease mitigation strategies and clinical care. Factors that showed a significant relationship in univariate analysis but were removed or deemed non-significant in multivariate analysis may have their roles explained by other significant factors. As a result, they do not appear to have an independent role in mortality. Overall, healthcare systems should consider a mitigation plans to address alcohol misuse and respiratory illnesses in patients at risk for iGAS.

Our study had several limitations: not all samples were sent to the reference lab, so not all *emm* types are known; there was no clear definition of alcohol misuse regarding the number of units consumed per week; deaths due to all-cause mortality within 7 days may not capture all iGAS deaths with longer complications; and data on antibiotic usage, which can affect outcomes, was not collected.

In conclusion, we found that, over eight years in Scotland case fatality was high, and strongly associated with a combination of clinical, demographical, and microbiological factors. Age-specific clinical and public health prevention and management interventions are required to reduce mortality. Greater understand of the pathophysiological basis of *emm* types association with higher mortality is required, and potentially could support future novel therapeutic and vaccination approaches. Training healthcare providers on the significance of these risk factors will ensure they can effectively identify and manage at-risk patients.

## Funding

This paper was funded by Wellcome (304666/Z/23/Z) and an NIHR Global Health Research Professorship (NIHR304311). The views expressed are those of the author(s) and not necessarily those of the NIHR or the Department of Health and Social Care. For the purpose of open access, the author has applied a CC BY public copyright licence to any Author Accepted Manuscript version arising from this submission.

## Ethic approval

The Public Health Scotland (PHS) is responsible to control infectious disease spread according to the National Health Service (Scotland) Act 1978. PHS must process data related to notifiable diseases and health risks without requiring individual patient consent, as outlined in the Public Health etc. (Scotland) Act 2008.

## Declaration of competing interest

The authors have no competing interests to declare.
